# Abdominal pain due to a lost guidewire: a case report

**DOI:** 10.4076/1757-1626-2-6680

**Published:** 2009-08-12

**Authors:** Reza Taslimi, Saeed Safari, Alireza Kazemeini, Ali Aminian, Ehsan Joneidi, Farnoosh Larti

**Affiliations:** 1 Department of Internal Medicine, Imam Khomeini Hospital, Tehran University of Medical SciencesTehranIran; 2 Department of General Surgery, Imam Khomeini Hospital, Tehran University of Medical SciencesTehranIran; 3 Department of Cardiology, Imam Khomeini Hospital, Tehran University of Medical SciencesTehranIran

## Abstract

A lost guidewire is a hazardous, yet completely preventable, and rare complication associated with central venous catheter insertion. Here, we report a case of a lost guidewire in a patient presented with persistent abdominal pain. The guidewire was retrieved completely during a surgical operation after the diagnosis had been confirmed by radiologic studies. Following some tips during insertion of a central venous catheter will help to prevent this mortal complication or at least, in rapid diagnosis of its loss. Interventional radiologic techniques are now readily used to retrieve a lost guidewire.

## Case presentation

A 28-year-old Iranian white man came to our emergency department (ED) with a 4 day history of abdominal pain, jaundice and fever. He had epistaxis since the day before admission. The patient was a heroin addict and also a known case of active hepatitis B for the previous 2 years, intermittently treated with lamivudine.

The patient was stable and not dehydrated. Low grade fever and generalized abdominal pain with mild tenderness had been detected on examination. No stigmata of chronic liver disease were found. Abdominal X-rays and ultrasound examination were normal. Baseline laboratory data revealed leukocytosis of 31,400/microL, hemoglobin of 12.1 g/dL, creatinine level of 9.0 mg/dL and urea level of 300 mg/dL. Urine analysis showed moderate leukocyturia. A diagnosis of acute renal failure (probably due to acute interstitial nephritis secondary to analgesic drugs that the patient had used to quit drug addiction) was made and emergent hemodialysis was planned. A femoral catheter was inserted by emergency department physician and there was no problem during the dialysis sessions.

After hemodialysis, the patient’s symptoms relieved to a little degree.

The patient’s abdominal pain recurred again just after a day. On the fourth day of admission, a drop in hemoglobin to the level of 7.5 g/dL was detected.

**Figure 1.**
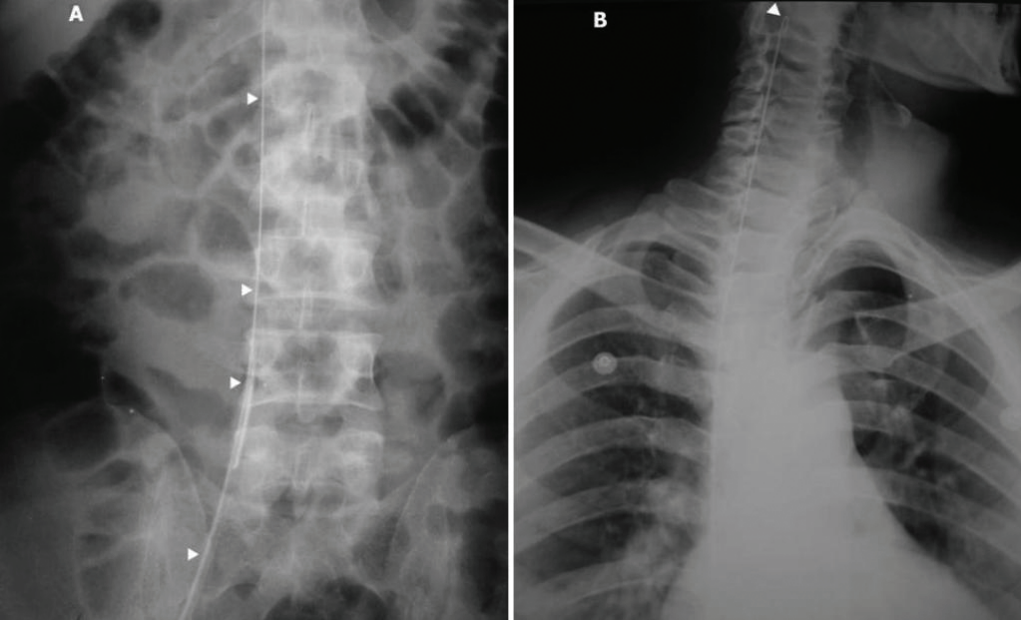
Plain abdominal X-ray (2nd day of admission) showed ileus and a linear density along the vertebral column (panel A, arrowheads). That linear density resembled a metallic guidewire and had a J-tipped end (panel B, arrowhead).

**Figure 2.**
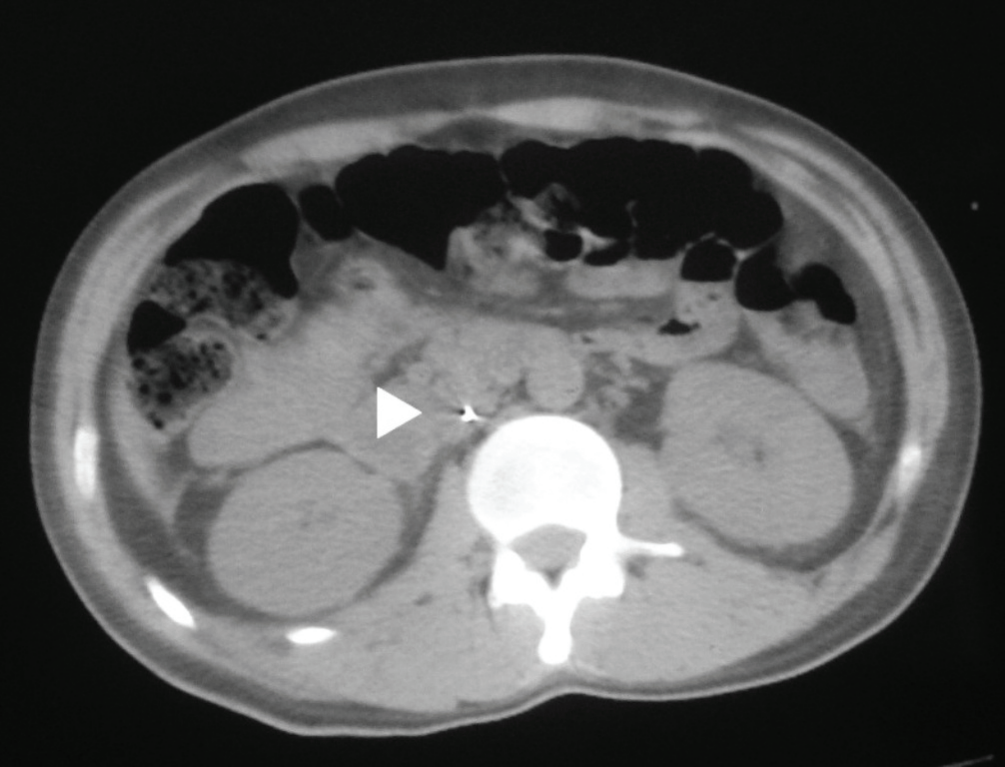
CT scan of the abdomen revealed a shiny density near the vertebral column (arrowhead).

On examination, nothing was found except for little bruising in the groin region and moderate generalized abdominal tenderness. New radiographic studies revealed ileus and a linear density along the vertebral column up to the patient’s neck (Figure 1); surprisingly enough, it seemed like a metallic guidewire that usually is used for inserting hemodialysis catheter. Because of contraindication for IV contrast injection, a non-contrast CT scan showed an unusual shiny density near the vertebral column and free fluid in the pelvis (Figure 2); no signs of hematoma or vascular injury were seen. With the impression of a forgotten guidewire and possible vascular injury (with hemoglobin drop and abdominal pain as signs for major vascular injury), the patient went to the operating room for an exploratory laparotomy. A laparotomy revealed some ascites and large non-pulsatile confined retroperitoneal hematoma in the pelvic area (zone III).

No major vascular injury was detected. According to the preoperative images that showed the location of guidewire tip in the patient’s neck, an incision was made anterior to the right sternocleidomastoid muscle. Jugular vein was exposed and venous control was taken and through a venotomy, the guidewire was extracted without any resistance. No change in the size of pelvic hematoma was noted during catheter removal. Post-operative course was uneventful; significant improvement in the patient’s general condition and abdominal pain ensued. Hemodialysis was continued via the inserted femoral catheter till the patient discharged home.

## Discussion

Percutaneous central venous catheter (CVC) insertion (for venous access or hemodialysis) is becoming a routine procedure in ICU settings and also emergency departments. However, complications (some of which are life-threatening) associated with this seemingly “minor” procedure make it very dangerous when performed by inexperienced physicians. Some of the most common complications are transient arrhythmias, arterial puncture (with hematoma or excessive bleeding), pneumothorax, hemothorax or hemomediastinum, air embolism, line infection and sepsis, and occasionally loss of a guidewire or catheter fragments [1,2]. The lost guidewire or catheter is the only complication that is completely avoidable with a mortality rate as high as 20% [3].

There are some reports of forgotten guidewire in the literature [2,4-6]. Most of these cases were not recognized immediately after catheter insertion nor after the post insertion control chest X-ray (CXR). Only a repeat CXR or a new sign or symptom (cardiac tamponade [6], sustained abdominal pain in our case) alarmed for this complication. This means that we are not thinking of this complication because it is not familiar to us. Informing very soon makes it possible to retrieve the guidewire in case that it is still inside the catheter (not travelling completely into the vascular bed) [2].

Reviewing the cases show that factors such as inexperienced physician, inadequate supervision by an experienced physician, busy and overtired medical staff (in our case, the responsible physician in emergency department performed the procedure at 4 A.M., at the end of a busy workday), and hastiness are associated with this type of human error [2,4,5].

Seldinger technique (inserting a catheter over a metallic and flexible J-tipped guidewire) is the most common technique used for CVC insertion. Some tips to avoid the complication when using this technique are recommended. First, the physician should check the catheter and guidewire carefully for any breakage before the procedure. Second, it is mandatory to hold the proximal end of the guidewire all the time during the procedure. Third, the physician should never push forward the guidewire when encountering a resistance. Instead, removal and inspection of the guidewire for any damage are recommended. Forth, always after performing the procedure it is better to check the catheter set for the presence of the guidewire. It must be there. Fifth, always after the procedure obtain a control CXR and watch it yourself for all possible complications.

Retrieving a missed guidewire or catheter fragments is now a routine angiographic intervention. Unless the patient is unstable or any concern exists about vascular damage, surgical intervention is the last choice. There are various angiographic techniques for percutaneous removal of intravascular foreign bodies, one of the most common is angiographic snaring [7]. Other devices like Dormia baskets, balloon catheters and grasping devices are also used. In our case, the patient’s hemoglobin drop convinced us not to choose the angiographic option, but to operate on the patient.

## Conclusion

Every physician who is trying to insert a CVC must be familiar with its complications, especially a lost guidewire. We have proposed some tips in order to prevent this complication. Checking the catheter set for the guidewire at the end of the procedure and taking a control CXR is mandatory. When you suspect a lost guidewire, immediate consulting with an expert surgeon and an expert interventional radiologist is the most prudent approach.

## Abbreviations

CVC: central venous catheter
CXR: chest X-ray
